# Exploring the potential of Bornean polypore fungi as biological control agents against pathogenic *Ganoderma boninense* causing basal stem rot in oil palm

**DOI:** 10.1038/s41598-023-37507-0

**Published:** 2023-06-26

**Authors:** Darwana Darlis, Mohamadu Boyie Jalloh, Clament Fui Seung Chin, Noor Khairani Mohamad Basri, Normah Awang Besar, Khairulmazmi Ahmad, Mohd. Rashid Mohd. Rakib

**Affiliations:** 1 Faculty of Sustainable Agriculture, Universiti Malaysia Sabah, 90000 Sandakan, Sabah Malaysia; 2 Faculty of Tropical Forestry, Universiti Malaysia Sabah, 88400 Kota Kinabalu, Sabah Malaysia; 3 Department of Plant Protection, Faculty of Agriculture, Universiti Putra Malaysia, 43400 Serdang, Selangor Malaysia

**Keywords:** Fungal pathogenesis, Pathogens

## Abstract

Basal stem rot due to a fungal pathogen, *Ganoderma boninense*, is one of the most devastating diseases in oil palm throughout the major palm oil producer countries. This study investigated the potential of polypore fungi as biological control agents against pathogenic *G. boninense* in oil palm. In vitro antagonistic screening of selected non-pathogenic polypore fungi was performed. Based on *in planta* fungi inoculation on oil palm seedlings, eight of the 21 fungi isolates tested (GL01, GL01, RDC06, RDC24, SRP11, SRP12, SRP17, and SRP18) were non-pathogenic. In vitro antagonistic assays against *G. boninense* revealed that the percentage inhibition of radial growth (PIRG) in dual culture assay for SRP11 (69.7%), SRP17 (67.3%), and SRP18 (72.7%) was relatively high. Percentage inhibition of diameter growth (PIDG) in volatile organic compounds (VOCs) in dual plate assay of SRP11, SRP17, and SRP18 isolates were 43.2%, 51.6%, and 52.1%, respectively. Molecular identification using the internal transcribed spacer gene sequences of SRP11, SRP17, and SRP18 isolates revealed that they were *Fomes* sp., *Trametes elegans*, and *Trametes lactinea,* respectively.

## Introduction

The African oil palm (*Elaeis guineensis* Jacq.) is an oil-producing crop cultivated primarily in tropical regions, especially in Indonesia and Malaysia, constituting 60% and 25% of the global vegetable oil supplies, respectively^1^. However, the large-scale cultivation of oil palm is seriously threatened by basal stem rot disease (BSR), a devastating disease mainly caused by *Ganoderma boninense*, a phytopathogen basidiomycete fungus^2,3^. Infected plants usually rot at the palm stem base, collapse, and eventually die. Basal stem rot infections has resulted in significant yield loss due to a reduction in the number of standing oil palm trees per hectare and productivity of the fresh fruit bunches^4,5^. Based on a study in three oil palm plantations located in Sabah, Malaysia, it was estimated that the yield loss due to the disease can be up to 68.73% in 12 months, which is estimated to be an average of USD4112.78 per year^6^.

To date, effective prophylactic and curative treatments are not yet available for the disease. Nevertheless, integrated disease management is highly recommended to minimise the economic loss through disease control methods, such as the use of biological control agents (BCAs), cultural practices, and chemical treatments^7,8^. In this respect, BCA is a sustainable practice for crop disease management since it is non-toxic and environmentally friendly^9,10^. Several BCA’s effects against *G. boninense* are shown to be promising. These include the ascomycete fungi such as *Trichoderma* spp.^11–13^ and *Hendersonia* sp.^14^, the bacteria *Pseudomonas fluorescens* and *Bacillus* sp.^7,15^, several rhizospheric and endophytic bacteria such as *Pseudomonas* spp. and *Burkholderia* spp.^16,17^, as well as the actinomycetes *Streptomyces* sp.^18^. Some of the criteria used for selection of a BCA is mainly based on their ability to compete with the pathogens for nutrients and space, parasitism, as well as the production of secondary metabolites, volatile organic compounds, and enzymes^19^.

However, the genetic variability of *G. boninense* due to basidiospore outcrossing has contributed to variability in the current BCA’s tolerance levels^20,21^. Consequently, there is a need to identify more BCA candidates to overcome this problem. The use of fungi from the order Polyporales, or also known as polypore fungi, which are distinguished by the formation of large fruiting bodies with pores or tubes on the underside, has been least explored to evaluate their potential as BCAs against *G. boninense*. In Malaysia, Naidu et al. first reported the in vitro inhibition of *G. boninense* using polypore fungi that is naturally found on oil palm trunks, namely *Pycnoporus sanguineus*, *Trametes lactinea*, and *Grammothele fuligo*^22^.

Due to the scarcity of research on polypore fungi as BCA against *G. boninense*, this study looked into more potential isolates, particularly those available in the rich Borneo rainforest that have not been reported before. The potential of Bornean polypore fungi as BCAs was thus evaluated in this study. The pathogenicity of the polypore fungi was first tested, based on *in planta* fungi inoculation of oil palm seedlings to identify the non-pathogenic polypores. This was followed by evaluation of the in vitro antagonistic effects of some selected non-pathogenic polypore fungi against *G. boninense*, based on dual culture and volatile organic compounds assays.

## Results

### Pathogenicity of polypore fungi

**Figure 1:**
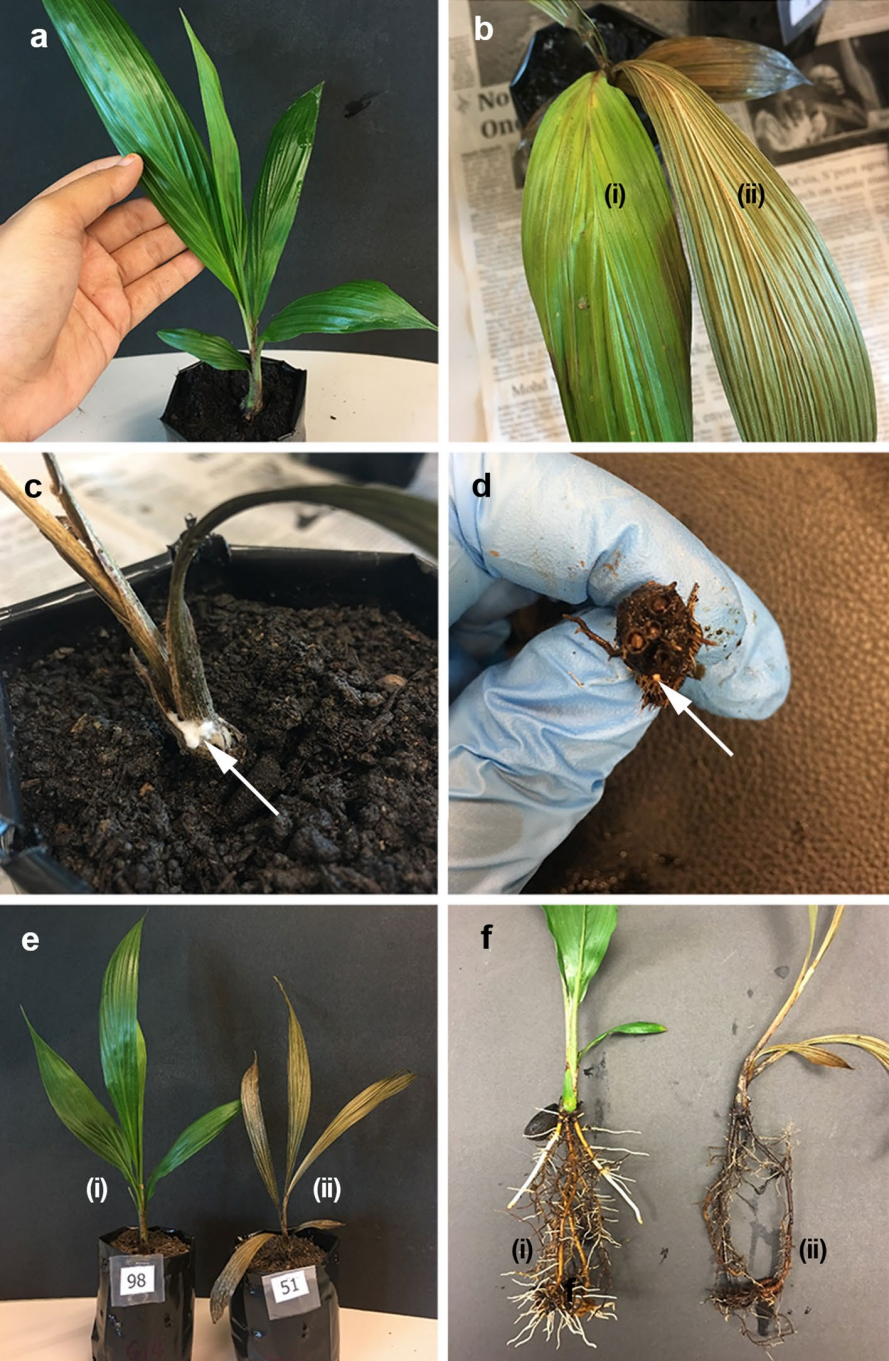
Disease signs and symptoms in oil palm seedlings at four weeks after the inoculation of polypore fungi, *in planta*. (**a**) Healthy or symptomless seedling. (**b**) Chlorotic (i) and necrotic (ii) leaves. (**c**) Fungal mass on an infected seedling (arrow). (**d**) Decayed primary roots (arrow). (**e**) Leaves of healthy (i) and infected (ii) seedlings. (**f**) Roots of healthy (i) and infected (ii) seedlings.

To distinguish between the pathogenic and non-pathogenic fungi, disease signs and symptoms in the tested oil palm seedlings, indicating successful infection of the polypore fungi were observed and recorded at week four post inoculation. The results are shown in Fig. 1. Among the 21 polypore fungi isolates tested, 12 of them caused infection with typical disease signs and symptoms, including the appearance of chlorotic leaves, necrotic leaves, fungal mass on the oil palm seedling bole, and necrotic roots after four weeks of incubation. The expressed disease signs and symptoms were similar in all the infected seedlings, regardless of the polypore fungi isolates tested. Uninoculated seedlings, seedlings inoculated with two non-pathogenic *Ganoderma lucidium* isolates and six other polypore fungi isolates remained symptomless throughout the experiment.

Table 1 depicts the degree of infection by *G. boninense* in oil palm seedlings based on disease incidence, disease severity, percentage of symptomatic leaves, and percentage of infected primary roots. Oil palm seedlings inoculated with the polypore fungi *G. lucidum* (GL01 and GL02), RDC06, RDC24, SRP11, SRP12, SRP17, and SRP18 showed no disease incidence and were healthy, similar to the uninoculated negative control. These fungi isolates were deemed non-pathogenic and were chosen for subsequent in vitro antagonistic assays.

**Table 1:** Isolates pathogenicity at four weeks after inoculation on oil palm seedlings based on the mean of visible symptoms (N = 5).

Isolate code	Mean ± standard error				Pathogenicity
	Disease incidence (%)	Disease severity index (%)	Percentage of symptomatic leaves (%)	Percentage of infected primary roots (%)	
Uninoculated	0.0	0.0 ± 0.0c	0.0 ± 0.0d	0.0 ± 0.0e	Non-pathogenic
G4	100.0	65.0 ± 10.0a	64.0 ± 9.3a	78.3 ± 9.7a	Pathogenic
GL01	0.0	0.0 ± 0.0c	0.0 ± 0.0d	0.0 ± 0.0e	Non-pathogenic
GL02	0.0	0.0 ± 0.0c	0.0 ± 0.0d	0.0 ± 0.0e	Non-pathogenic
RDC06	0.0	0.0 ± 0.0c	0.0 ± 0.0d	0.0 ± 0.0e	Non-pathogenic
RDC07	40.0	20.0 ± 12.2bc	18.0 ± 11.1cd	8.0 ± 4.9e	Pathogenic
RDC14	60.0	20.0 ± 9.4bc	24.0 ± 9.9bcd	33.3 ± 13.9cd	Pathogenic
RDC15	80.0	20.0 ± 5.0bc	46.0 ± 16.0ab	53.3 ± 16.2bc	Pathogenic
RDC24	0.0	0.0 ± 0.0c	0.0 ± 0.0d	0.0 ± 0.0e	Non-pathogenic
RDC26	60.0	20.0 ± 9.4bc	20.0 ± 9.4cd	15.3 ± 7.4de	Pathogenic
RDC30	40.0	10.0 ± 6.1c	5.0 ± 2.0cd	6.7 ± 6.7e	Pathogenic
SRP01	100.0	60.0 ± 10.0a	65.0 ± 12.71a	76.7 ± 14.5a	Pathogenic
SRP02	20.0	0.0 ± 0.0c	0.0 ± 0.0d	5.0 ± 2.5e	Pathogenic
SRP06	80.0	35.0 ± 10.0b	28.0 ± 11.6bc	10.7 ± 6.6de	Pathogenic
SRP08	40.0	15.0 ± 10.0bc	13.0 ± 8.3cd	9.0 ± 5.6e	Pathogenic
SRP11	0.0	0.0 ± 0.0c	0.0 ± 0.0d	0.0 ± 0.0e	Non-pathogenic
SRP12	0.0	0.0 ± 0.0c	0.0 ± 0.0d	0.0 ± 0.0e	Non-pathogenic
SRP14	80.0	60.0 ± 12.7a	54.0 ± 13.3a	58.3 ± 12.9ab	Pathogenic
SRP17	0.0	0.0 ± 0.0c	0.0 ± 0.0d	0.0 ± 0.0e	Non-pathogenic
SRP18	0.0	0.0 ± 0.0c	0.0 ± 0.0d	0.0 ± 0.0e	Non-pathogenic
SRP23	80.0	15.0 ± 6.1bc	14.0 ± 5.8 cd	7.3 ± 4.5e	Pathogenic
SRP29	80.0	20.0 ± 5.0 bc	21.0 ± 6.4 cd	23.3 ± 6.1de	Pathogenic

Mean values followed by different letters within columns indicate significant difference at p ≤ 0.05 via Tukey’s test.

The positive control (G4), SRP01, and SRP14 are highly pathogenic, with 80% to 100% disease incidence, 60% to 64% disease severity, 54% to 64% symptomatic leaves, and 58.3% to 78.3% infected primary roots in the tested oil palm seedlings. Meanwhile, RDC14, RDC15, RDC26, SRP06, SRP23, and SRP29 were moderately pathogenic, with disease incidence ranging from 60 to 80%, disease severity ranging from 15 to 20%, symptomatic primary leaves ranging from 14 to 46%, and infected primary roots ranging from 7.3% to 53.3%. Oil palm seedlings inoculated with RDC07, RDC30, SRP02, and SRP08 exhibited the least infection. The disease incidence, disease severity, symptomatic leaves, and infected primary roots for these isolates were 20–40%, 10–20%, 5–18%, and 5.0–9.0%, respectively. Further, Pearson’s correlation analysis revealed that all dependent variables measured were positively correlated between each other, where they recorded high coefficients and, r values ranging from 0.80 to 0.97 at significance level of p < 0.001 (Table 2).

**Table 2:** Pearson’s correlation analysis between variables for disease signs and symptoms in oil palm seedlings (N = 110).

Variable	Disease severity	Symptomatic leaves	Infected primary roots
Disease incidence	0.86**	0.88**	0.80**
Disease severity		0.95**	0.90**
Symptomatic leaves			0.97**

**Significantly correlated at p ≤ 0.01.

### In vitro antagonistic assays

The antagonistic effect exerted by each non-pathogenic polypore fungi against *G. boninense* was evaluated through in vitro dual culture and VOCs dual plate assays. Table 3 shows the in vitro dual culture and VOCs dual plate assays of the eight selected non-pathogenic polypore fungi tested against *G. boninense* (G4). Based on their dual culture assay mean values, GL01 and SRP12 were weak antagonists (21–40%), while GL02, RDC06, and SRP02 were moderate antagonists (41–60%), and RDC24, SRP11, SRP17, and SRP18 were strong antagonists (61–80%). In the VOCs dual plate assay, RDC24 was a very weak antagonist (1–20%), while GL02, RDC06, SRP02, and SRP12 were weak antagonists (21–40%), and GL01, SRP11, SRP17, and SRP18 were moderate antagonists (41–60%).

**Table 3:** Mean percentage inhibition of radial growth (PIRG) and percentage inhibition of diameter growth (PIDG) of the individual isolates against *G. boninense* in in vitro culture assays (N = 3).

Isolate code	Mean ± standard error	
	PIRG in dual culture assay (%)	PIDG in volatile organic compounds dual plate assay (%)
GL01	37.0 ± 3.2e	40.8 ± 5.1b
GL02	52.7 ± 1.0d	26.8 ± 1.4cd
RDC06	54.5 ± 1.0d	35.2 ± 4.9bc
RDC24	61.2 ± 1.2c	2.3 ± 0.9e
SRP11	69.7 ± 1.2ab	43.2 ± 0.5ab
SRP12	39.4 ± 1.6e	23.9 ± 0.8d
SRP17	67.3 ± 1.8b	51.6 ± 3.3a
SRP18	72.7 ± 1.0a	52.1 ± 2.8a

Mean values followed by different letters within columns indicate significant difference at p ≤ 0.05 via Tukey’s test.

**Figure 2:**
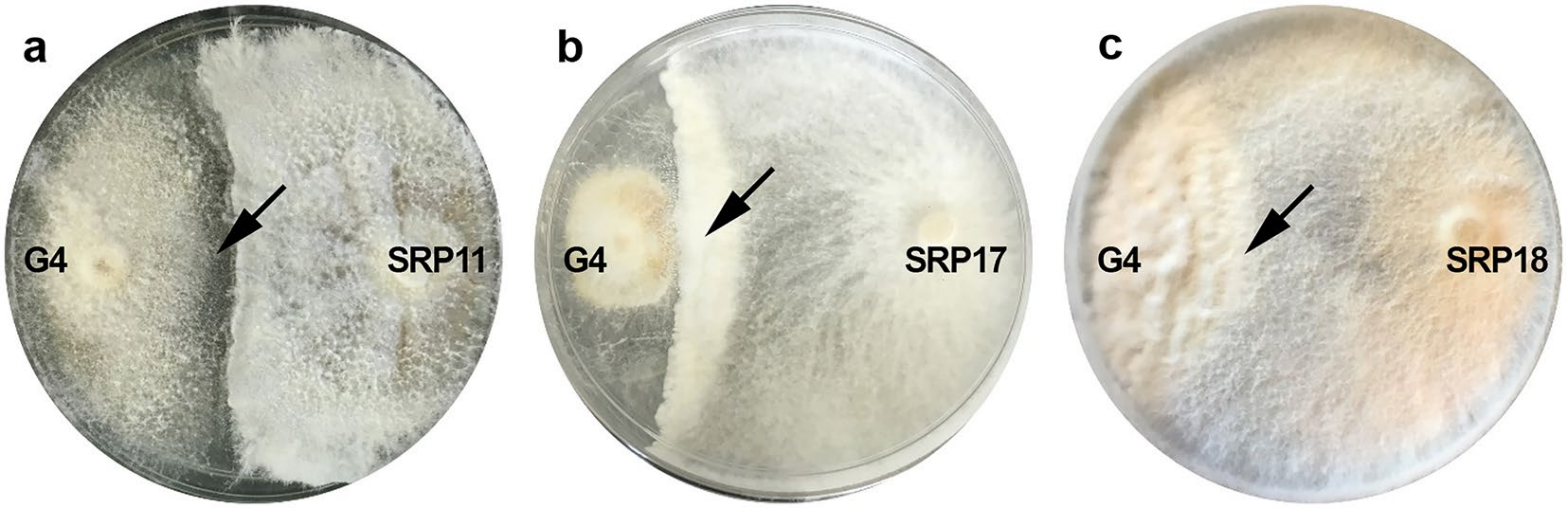
In vitro dual culture assay showing inhibition of the potential antagonists against *G. boninense* (G4), where the inhibition is indicated by arrows. (**a**) Antagonism of SRP11 against *G. boninense* (G4). (**b)** Antagonism of SRP17 against *G. boninense* (G4). **c** antagonism of SRP17 against *G. boninense* (G4).

The ability of the fungi to inhibit *G. boninense* in the dual culture and VOCs dual plate assays were not significantly correlated (p = 0.38, r = 0.33). Also, SRP11 and SRP18 recorded the highest PIRG in the dual culture assay at 67.7% and 72.7%, respectively, followed by SRP17 (67.3%). Similarly, SRP11, SRP17, and SRP18 recorded the highest PIDG in the VOCs dual plate assay at 43.2%, 51.6%, and 52.1%, respectively. Therefore, the three isolates (SRP11, SRP17, and SRP18) were selected for further investigation as potential candidates of BCAs against *G. boninense* in oil palm (Fig. 2). Based on the ITS sequences, the isolates of SRP11, SRP17, and SRP18 were identified as *Fomes* sp., *Trametes elegans,* and *Trametes lactinea*, respectively, with 99 – 100% homology with the deduced nucleotide fragment sequences searched using BLAST (Table 4).

**Table 4:** Identity of the potential polypore fungi as biological control agents based on the similarity between samples sequences and the sequences in GenBank.

Isolate code	Accession number (NCBI)	Identity	ITS fragment length (base pairs)	Homology (%)	BLAST bit score
SRP11	OK643764.1	*Fomes* sp.	644	100	1190
SRP17	LC120834.1	*Trametes elegans*	621	99	1053
SRP18	JX082368.1	*Trametes lactinea*	619	100	1114

## Discussion

Typical *Ganoderma* infection was observed on the oil palm seedlings. The infection was initiated at the root since the roots and inoculum source were in direct contact. The infection later progressed to the stem bole, mainly through the inner thin-walled cortex. Then, foliar symptoms (chlorosis and necrosis) appeared as a result of the damaged internal stem bole or disruption of the xylem and phloem resulting in the water and nutrients required for the seedlings not being sustained, which caused progressive desiccation of the leaves from the oldest to the youngest. The infected seedlings eventually died, either with or without visible fungal masses on the seedlings above ground^23,24^. Furthermore, the *in planta* infection of oil palm seedlings in this study adopted from Purnamasari et al. showed similar disease progression over four weeks^25^, compared with those infected using the technique of placing rubber wood block with *Ganoderma* as inoculum on older oil palm seedlings, i.e. 3-months-old, and data collection carried out over six months^23,24,26^.

The different levels of aggressiveness of the polypore fungi tested could be related to the modes of nutrition acquisition strategies during fungal pathogen infection on the plants, which have traditionally been classified into biotrophic and necrotrophic types of parasitism^27^. The highly pathogenic isolates identified in this study, SRP01 and SRP14, as well as the positive control *G. boninense* (G4), were necrotrophs, which are facultative parasites that derive energy from a living host, kill the host's cells, and then live saprophytically on the dead remains^28^. According to Idris et al.^29^ and Castillo et al.^30^, three species of pathogenic *Ganoderma*, namely, *G. boninense*, *G. zonatum*, and *G. miniatocinctum* were associated with BSR in oil palm. Other species, such as *G. tornatum*, *G. applanatum*, *G. lucidum*, *G. pfeifferi,* and *G. philippi,* were not pathogenic^31^. Meanwhile, the least infection in the isolates RDC07, RDC30, SRP02, and SRP08 could be related to biotrophic facultative saprophytic parasitism, where the fungi derive energy from living cells, have very complex nutrient requirements, and do not kill the host plants rapidly^28,32^.

The polypores, RDC24, SRP11, SRP17, and SRP18 in this study demonstrated strong antagonistic activity against *G. boninense* (G4) based on the dual culture assay, with a PIRG value of 61.2% to 72.7%. The findings in this study are similar to that reported by Naidu et al., where the in vitro PIRG value of *G. boninense* using the same assay was between 70 and 80%^22^. The fungi *Pycnoporus sanguineus*, *Trametes lactinea*, and *Grammothele fuligo* inhibited the in vitro growth of *G. boninense* by 84%, 82%, and 81%, respectively. Furthermore, another study by Naidu et al. also reported that in vitro antagonism of *G. boninense* using the same assay was between 73 and 85%, where *Neonothopanus nambi* exhibited high antagonism^33^. In general, the inhibition of *G. boninense* could be due to the predominance of BCAs against the pathogen due to competition for the available nutrients and space, parasitism, the secretion of antibiotics, or the production of secondary and volatile metabolites, and enzymes^7,19,34^. Non-volatile metabolites (e.g., alkaloids, flavonoids and fatty acids), and volatile metabolites (e.g., aromatic metabolites, terpenes, polyketides, butenolides and pyrones) are among the metabolites that have antifungal activity against fungi^35,36^. These metabolites may inhibit ergosterol synthesis (the main fungal sterol) and macromolecular synthesis, and be toxic to phytopathogens^37^. In addition, hydrolytic enzymes such as the chitinases produced by BCAs are able to degrade the fungal cell wall of phytopathogens, which further increases the permeability of the cell wall to subsequent entry of antifungal metabolites. Furthermore, several proteases from BCAs have been reported to act on the phytopathogens’ enzymes and impair their functions^19^. Several studies reported that the secondary metabolites released by *Trametes* spp. showed antifungal properties with inhibitory effects on complex phytopathogenic fungi such as *G. boninense*^38,39^. Moreover, it was reported that the fruiting bodies of *Trametes* spp. contained phenolic and flavonoid compounds and other chemicals, such as saponin, tannin, and alkaloid, giving them antifungal properties^39,40^.

Although the potential of *Trametes lactinea* as a BCA to suppress *G. boninense* in oil palm has been previously reported^22,29^, there is still a lack of information available on the role of *Trametes* species as BCAs against crop diseases. In particular, *Trametes versicolor* was the most dominant species of this genus in controlling the tomato pathogens *Clavibacter michiganensis*, *Ralstonia solanacearum,* and *Leveillula taurica*^41,42^ (Table 5). However, the role of *T. elegans* and *Formes* sp. as BCAs in controlling *G. boninense* or other phytopathogens remains unknown.

**Table 5:** The role of *Trametes* sp. as biological control agents against phytopathogens.

Fungi	Pathogen	Disease and crop	*References
*T. lactinea*	*Ganoderma boninense*	Basal stem rot in oil palm	^20,24^
*T. versicolor*	*Clavibacter michiganensis*	Bacterial canker in tomato	^37^
*T. versicolor*	*Ralstonia solanacearum*	Bacterial wilt in tomato	^37^
*T. versicolor*	*Leveillula taurica*	Powdery mildew in tomato	^38^

The Bornean non-pathogenic polypore fungi discovered in this study need to be further characterized, such as by evaluating their metabolite production using liquid chromatography-mass spectrometry (LC–MS) and gas chromatography-mass spectrometry (GC–MS), for better understanding of their roles in exerting antagonism towards *G. boninense*. Furthermore, *in planta* suppression of *G. boninense* infection utilizing the possible antagonists discovered, either at seedling, immature, or mature stages of oil palm, will require longer-term observation and data collection to corroborate the findings.

## Materials and methods

### Pathogenicity of polypore fungi

A total of 150 germinated oil palm seeds (a cross between Deli dura and AVROS pisifera) were acquired from Sawit Kinabalu Balung, Tawau, Sabah, Malaysia (4.2667° N, 117.9631° E), a commercially certified oil palm seed producer. The germinated oil palm seeds were sown in three planting pot trays (50 pots per tray) containing topsoil. The seedlings were grown in a rain-shelter facility at the Faculty of Sustainable Agriculture of Universiti Malaysia Sabah (5.9300° N, 118.0112° E) for three months, with standard nursery management practices^43^. These seedlings were used for the pathogenicity experiment.

The pathogenicity of 21 polypore fungi initially isolated by Darwana et al.^44^ was used in this study, to evaluate whether they were pathogenic or non-pathogenic towards oil palm. The fungi were isolated from the basidiocarp using potato dextrose agar (PDA) containing 50 mg L^−1^ streptomycin to prevent the growth of bacteria^45^. The pure cultures of the fungi were confirmed as dikaryotic basidiomycetes based on the presence of hyphal septa and clamp connections under microscopic observation. The fungi used in this study were obtained from its stock culture on PDA slant stored in 4 °C temperature.

The positive control comprised of *G. boninense* (G4) isolated from a BSR-infected oil palm, while two *G. lucidum* isolates (GL01 and GL02) served as the non-pathogenic internal control. The taxonomic identity of G4, GL01, and GL02 had been previously verified based on their genomic DNA fragment sequences (Supplementary Appendix S1). Other polypore fungi samples were collected from the Rainforest Discovery Centre (RDC; 5.8746° N, 117.9436° E) and Sandakan Rainforest Park (SRP; 5.8511° N, 118.0642° E) in Sandakan, Sabah, Malaysia. Samples were denoted by their local origin, i.e., RDC or SPC, followed by sample number.

Fungal inoculums preparation and oil palm seedling inoculation were prepared according to the method of Purnamasari et al., with minor modifications^25^. Specifically, a seven-day-old active mycelial plug (10 mm in diameter) was cultured in an Erlenmeyer flask containing 150 mL of potato dextrose broth (PDB) and incubated at 28 °C for five days on a shaking incubator at 150 rpm. The mycelia were filtered and transferred into a beaker containing 200 mL of sterile distilled water and 0.002% Tween 20. The mycelia was then blended using a 70% ethanol-sanitised handheld blender (Panasonic MX-SS40) for three minutes. The concentration of mycelia was adjusted to 10^5^ fragments mL^−1^ using a hemocytometer.

Oil palm seedlings were carefully removed from the pot tray, and excessive soil was loosened from the roots before soaking in the fungi inoculum for 30 min. Uninoculated seedlings soaked in sterile distilled water served as the control. All seedlings were transplanted into polybags (15 cm × 30 cm) containing 600 g of sterilised topsoil with five replications per treatment, yielding 110 experimental units. They were arranged based on a completely randomised design (CRD) in an environmentally regulated laboratory at 25–28 °C temperature, 70–80% relative humidity, and sufficient sunlight. The seedlings were watered twice daily with sterile distilled water to maintain soil moisture.

The presence of the pathogen (disease sign) and disease symptoms were recorded four weeks post inoculation. These disease signs and symptoms included disease incidence, disease severity, the total number of leaves, the number of symptomatic leaves, the total number of primary roots, and the number of infected primary roots^23^. The visually observable symptoms of infected seedlings was based on numerical values of disease class ranging from 0 to 4 (Table 6)^46^. The numerical values were transformed to a percentage of disease severity using Eq. (1). The procedures involving plant samples were performed in accordance with the relevant guidelines and regulations, including autoclaving the samples to eliminate all possible biological contamination before disposal of the samples.

1 $${\text{Disease}}\;{\text{severity}}\;(\% ) = \frac{{{\text{Disease}}\;{\text{class}}\;{\text{of}}\;{\text{seedling}}}}{{{\text{The}}\;{\text{highest}}\;{\text{disease}}\;{\text{class}}}} \times 100$$

**Table 6:** Numerical disease classes and their corresponding symptoms on oil palm seedlings.

Class	Symptoms
0	Healthy plants
1	Slight chlorotic spots on the leaves
2	Chlorosis on 1–2 leaves
3	Chlorosis on > 2 leaves
4	Dead plants

### In vitro antagonistic dual culture assays

The antagonistic effect of selected non-pathogenic polypore fungi against *G. boninense* was evaluated using the dual culture assay of Rahman et al.^47^. Mycelia plugs (8 mm in diameter) of the potential antagonist and pathogen (G4) were inoculated 20 mm away from the edge of a Petri dish containing PDA (90 mm diameter) on opposite sides. A PDA plate without an antagonist served as the control. The experiment was performed with three replications, arranged in a CRD, incubated for seven days, at 28 °C, and in darkness. The percentage inhibition of radial growth (PIRG) was calculated using Eq. (2)^48^.

2 $${\text{Percentage}}\;{\text{inhibition}}\;{\text{of}}\;{\text{radial}}\;{\text{growth}}\;(\% ) = \frac{{{\text{R1}} - {\text{R2}}}}{{{\text{R1}}}} \times 100$$

where, R1 is the colony radius of *G. boninense* (G4) isolates in the control plate and R2 is the colony radius of *G. boninense* (G4) isolate in the dual culture assay. The inhibition zone formed between the colonies was also observed. The antagonistic effect of each isolate against the growth of the *G. boninense* (G4) was classified as very weak (1–20%), weak (21–40%), moderate (41–60%), strong (61–80%), and very strong (81–100%) based on their PIRG values.

### In vitro antagonistic volatile organic compounds (VOCs) dual plate assays

**Figure 3:**
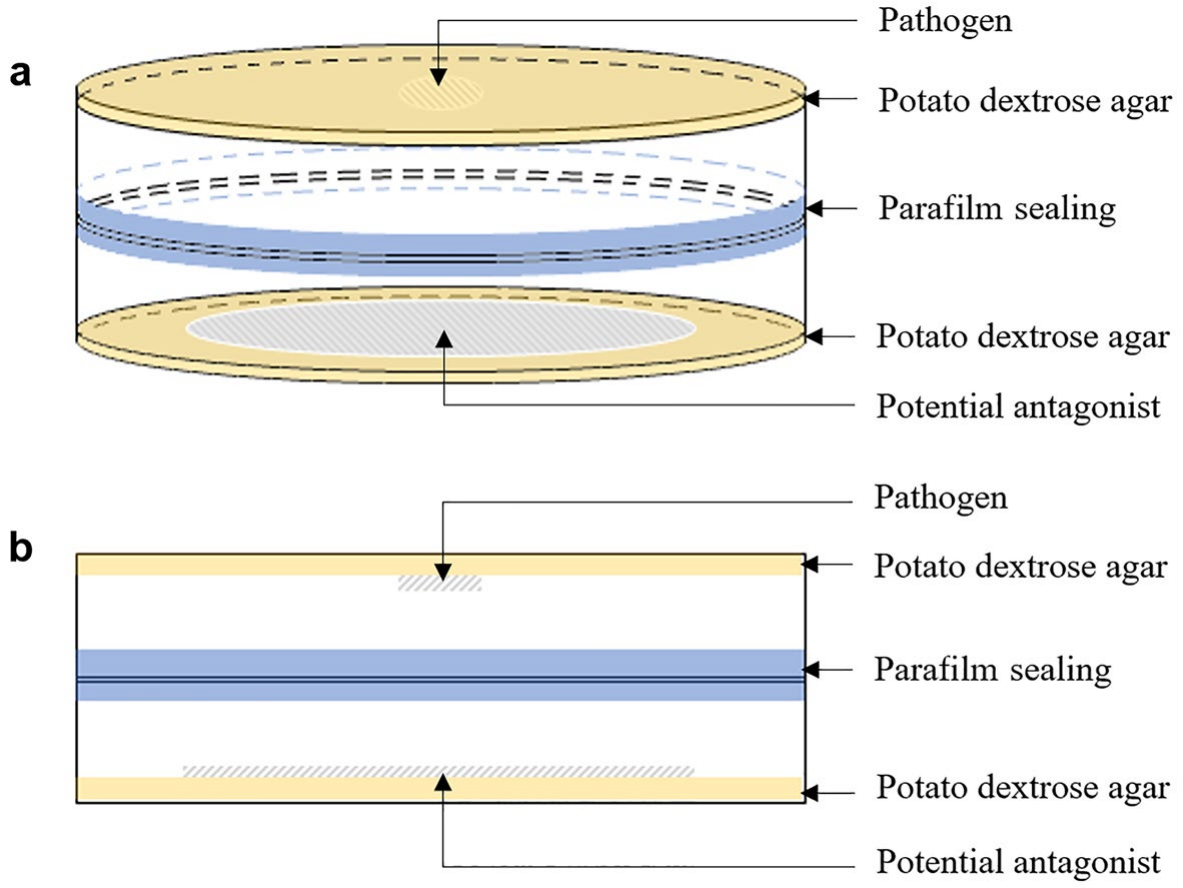
Schematic diagram of the volatile organic compound dual plate assay setup. (**a**) Three-dimensional view of the experimental unit. (**b**) Side view of the experimental unit.

An assay of VOCs was performed to evaluate the inhibitory potential of the non-pathogenic polypore fungi against *G. boninense* (G4) via the production of volatile inhibitory organic compounds^49^. The dual plate assay was performed according to Rajani et al.^50^. A mycelia plug (8 mm in diameter) of the potential antagonist was inoculated at the centre of a Petri dish containing PDA (9 cm diameter), incubated for seven days, at 28 °C, and in darkness. Then, the similar-sized mycelial plug of the pathogen, *G. boninense* (G4), was inoculated onto another PDA plate placed inversely over the PDA plate containing the seven-day-old antagonist, and sealed with parafilm. The VOCs from the antagonist were allowed to fumigate the pathogen. Plates with *G. boninense* without antagonists served as controls. The setup of the experiment is shown in Fig. 3, and they were arranged in CRD with three replications. The percentage inhibition of diameter growth (PIDG) was calculated using Eq. (3)^51^.

3 $${\text{Percentage}}\;{\text{inhibition}}\;{\text{of}}\;{\text{diameter}}\;{\text{growth}}\;(\% ) = \frac{{{\text{D1}} - {\text{D2}}}}{{{\text{D1}}}} \times 100$$

where, D1 is the colony diameter of G4 isolate in the control plate, and D2 is the colony diameter of G4 isolate exposed to the antagonist colony. The same inhibition classifications as that for the dual culture assay were adopted.

### Statistical analysis

Analysis of variance (ANOVA) was performed, and the means were compared using Tukey’s test at a significance level of 0.05. The relationships between the variables were evaluated using Pearson’s correlation analysis. Prior to the analysis, the statistical methods were validated in accordance with Zar^52^. All statistical analysis was performed using the Statistical Analysis System software (SAS version 9.4, North Carolina State University, USA).

### Molecular identification of the potential biological control agents

The genomic DNA (gDNA) of the potential BCAs was extracted from their pure cultures using a commercial kit (Norgen Biotek Corp., Canada), following the manufacturer’s protocol. The fungal internal transcribed spacer (ITS) gene was amplified using the polymerase chain reaction (PCR) with forward universal primer ITS1 (5′-TCCGTAGGTGAACCTGCGG-3′) flanking 5′-M13F (5′-GTAAAACGACGGCCAGT-3′), and reverse universal primer ITS4 (5′-TCCTCCGCTTATTGATATGC-3′) flanking 5′-M13R-pUC (5′-CAGGAAACAGCTATGAC-3′)^53^. The PCR amplifications were carried out in a 25 μL total reaction volume containing 1 μL gDNA, 0.5 pmol of each primer, deoxynucleotides triphosphates (dNTPs, 200 μM each), a 0.5 U DNA polymerase, PCR buffer, and water. Amplification was performed with an initial denaturation step (98 °C for 2 min), followed by 25 cycles of annealing and extension (98 °C for 15 s; 60 °C for 30 s; 72 °C for 30 s), and a final extension (72 °C for 10 min). The PCR products (1 μL) were run on 1% TAE agarose (agarose with a buffer solution containing a mixture of Tris base, acetic acid and EDTA) at 100 V for 60 min, which yielded 600 to 650 base pairs were purified using standard methods^54^. Sequencing of the purified PCR products was performed using a BigDye® Terminator v3.1 Cycle Sequencing Kit (Applied Biosystems, USA). The sequence was used to find the homologous sequence via the basic local alignment search tools (BLAST) in the National Center for Biotechnology Information (NCBI) website (https://www.ncbi.nlm.nih.gov/).

## Conclusion

Among the 21 polypore fungi isolates tested, eight isolates (GL01, GL02, RDC06, RDC24, SRP11, SRP12, SRP17, and SRP18) were non-pathogenic to oil palm seedlings. The seedlings appeared symptomless and similar to the uninoculated control. In vitro, antagonistic assays against *G. boninense* revealed that the PIRG value for the dual culture assay of SRP11, SRP17, and SRP18 were 69.7%, 67.3%, and 72.7%, respectively. Meanwhile, the PIDG value for the VOCs dual plate assay of SRP11, SRP17, and SRP18 were 43.2%, 51.6%, and 52.1%, respectively. This study successfully identified three polypore fungi; *Fomes* sp. (SRP11), *T. elegans* (SRP17) and *T. lactinea* (SRP18) as potential fungal BCAs against *G. boninense*. However, additional analysis, characterization, and validation assays are required to further justify and confirm their potential as efficient BCAs against *G. boninense*.

## Supplementary Information

Supplementary Information.

## Data Availability

All data generated or analyzed during this study are included in this published article and its supplementary information files.
