# Supplementary material for: Exploring the potential of Bornean polypore fungi as biological control agents against pathogenic Ganoderma boninense causing basal stem rot in oil palm
Source: Sci Rep. 2023 Jun 26;13:10316. doi: 10.1038/s41598-023-37507-0 (PMC10293268; doi:10.1038/s41598-023-37507-0)
Supplement: Supplementary file 1 — Supplementary Information. [file 41598_2023_37507_MOESM1_ESM.pdf]

Appendix S1. Identity of the isolates G4, GL01, and GL02 based on the similarity between samples sequences and the sequences in GenBank.

Isolate code	Accession number (NCBI)	Identity	ITS fragment length (base pairs)	Homology	BLAST bit score
G4	KX092000.1	*Ganoderma boninense*	655	98.92%	1085
GL01	JQ520182.1	*Ganoderma lucidum*	636	99.83%	1098
GL02	KT906368.1	*Ganoderma lucidum*	638	99.83%	1098
